# Mycophenolate mofetil and telmisartan for the treatment of proteinuria secondary to minimal change disease podocytopathy in a dog

**DOI:** 10.1111/jvim.16534

**Published:** 2022-09-24

**Authors:** Victoria Travail, Rachel E. Cianciolo, Kerry Peak, Andrea Di Bella

**Affiliations:** ^1^ Southern Counties Veterinary Specialists, Internal Medicine Forest Corner Farm Hampshire United Kingdom; ^2^ College of Veterinary Medicine, Veterinary Biosciences The Ohio State University Columbus Ohio USA; ^3^ Southern Counties Veterinary Specialists Hampshire United Kingdom

**Keywords:** kidney, nephrology, nephrotic syndrome, renal biopsy, renal/urinary tract

## Abstract

A 3‐year‐old entire female Springer Spaniel, with a previous diagnosis of meningoencephalitis of unknown origin diagnosed 2 years before presentation and treated with long term administration of prednisolone, developed proteinuria. Laboratory findings revealed hypoalbuminemia, hypercholesterolemia, and proteinuria. Further investigations excluded underlying causes. Renal biopsies were performed. The glomeruli and the tubulointerstitial compartment did not show any anomalies on light microscopy and immunofluorescence staining did not reveal abnormalities. Transmission electron microscopy revealed moderate podocyte injury consisting of foot process effacement and microvillus transformation of the cytoplasm. The dog was diagnosed with primary minimal change disease of the podocytes and treated with telmisartan and mycophenolate mofetil. Abnormalities of serum albumin, cholesterol, and proteinuria resolved within 4 weeks. Minimal change disease has been reported in dogs, but this is a case report of proteinuria secondary to minimal change disease successfully treated with mycophenolate mofetil and telmisartan.

**Abbreviations**
ALP: alkaline phosphatase
BUN: blood urea nitrogen
MCD: minimal change disease
MUO: meningoencephalitis of unknown origin
NIBP: noninvasive blood pressure
NS: nephrotic syndrome
UPC: urine protein creatinine ratio
USG: urine specific gravity

## 1 INTRODUCTION

The diagnosis of minimal change disease (MCD) podocytopathy is becoming more common in human medicine^1^, ^2^, ^3^, ^4^; however, it has only been sporadically described in veterinary medicine.^5^, ^6^, ^7^, ^8^, ^9^, ^10^ Patients with MCD can be asymptomatic in the early stages of the disease; however, MCD has been associated with acute kidney injury in 20% to 25% of adults,^11^ nephrotic syndrome (NS) in 15% of adults, and up to 70% to 90% of children with MCD develop idiopathic NS.^2^, ^12^ These conditions often present with proteinuria, hypoalbuminemia, hypercholesterolemia, presence of edema or ascites, and thrombus formation in humans.^2^, ^3^ The consequences of this condition can be life threatening so it is important to obtain an early diagnosis. Histopathology is necessary to diagnose MCD. Recognition of abnormal podocytes by electron microscopy is paramount for the diagnosis as light microscopy usually reveals no abnormalities.^2^, ^10^, ^13^, ^14^, ^15^ In veterinary medicine, MCD has been reported mainly through case reports or linked with the use of receptor protein kinase (c‐kit) inhibitors^7^ and in dogs experimentally infected by *Ehrlichia canis*.^5^ The disease is well documented in humans where it is believed to be immune‐mediated in the majority of cases; however, there are likely different subgroups of pathogenesis.^1^, ^2^, ^3^ This case report discusses a dog successfully treated for proteinuria secondary to minimal change disease podocytopathy with mycophenolate mofetil and telmisartan.

## 2 CASE HISTORY

A 3‐year and 11‐month‐old entire female Springer Spaniel dog was referred for further investigations of severe proteinuria. The dog had a history of meningoencephalitis of unknown origin (MUO) which was diagnosed when she was 9 months old and was treated with prednisolone. A month before presentation, the dog had a relapse of MUO, and had been receiving prednisolone 0.3 mg/kg per os once a day (Prednidale; Dechra, Skipton, UK) since that time. At the follow‐up neurologic examination, routine blood tests and urinalysis were performed and revealed proteinuria (urine protein creatinine ratio (UPC) 5.9 [ref <0.5]). Two subsequent UPC measurements were performed and documented persistent proteinuria. The urine had been tested on multiple occasions before this occasion and proteinuria had not been documented previously. On presentation the dog was bright, alert, and responsive. The physical examination revealed a mild strabismus. The rest of the vital signs were within normal limits and the body condition score was 4/9. Mean systolic noninvasive blood pressure (NIBP) was 154 mm Hg.

Hematology results did not detect abnormalities and the serum biochemistry revealed hypoalbuminemia (23 g/L [ref 24‐40]), hypercholesterolemia (9.8 mmol/L [ref 3.8‐7]) and elevation of alkaline phosphatase activity (ALP; 139 U/L [ref 0‐120]). Antibody testing for *Borrelia* spp., *Ehrlichia* spp., *Dirofilaria* spp., and *Anaplasma* spp. was negative. Antithrombin III was within reference interval (78% [ref 65‐145]). Coagulation profile (PT, aPTT, fibrinogen, and D‐Dimers) did not detect abnormalities. Buccal mucosal bleeding time was within normal range. Urinalysis revealed a urine specific gravity (USG) of 1.014. Abnormalities were not detected on urine sediment examination and UPC was 7.4 (ref <0.5). Bacterial culture of the urine was negative. Body cavity imaging (thoracic radiographs and abdominal ultrasound) was performed and did not reveal abnormalities.

**FIGURE 1:**
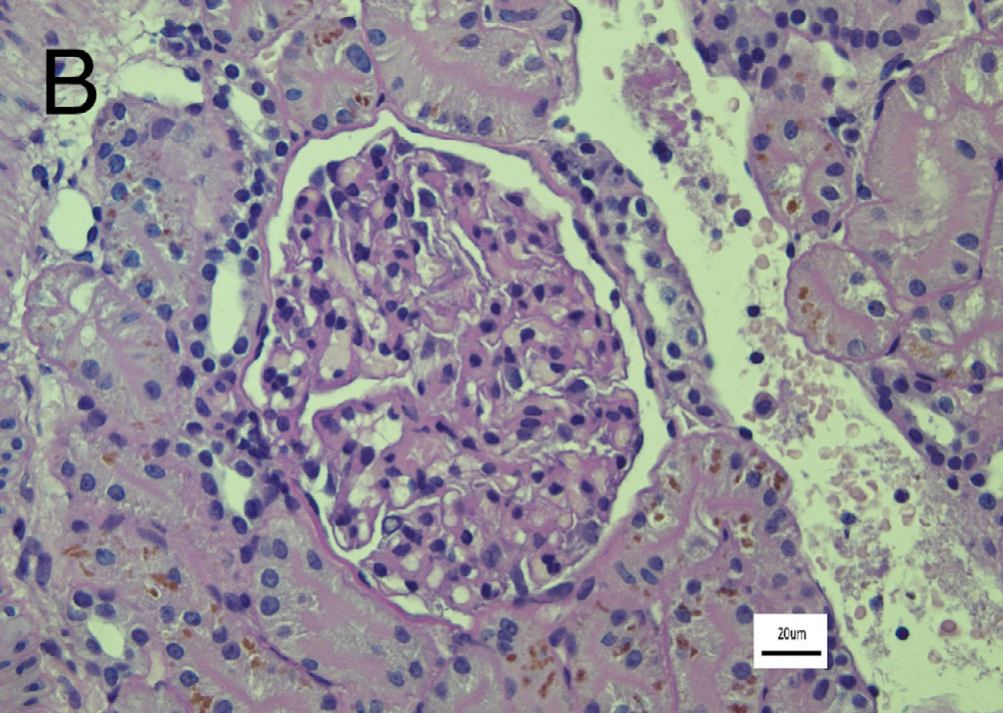
Light microscopy image of 11 glomerulus with Periodic Acid Schiff stain in a 3‐year‐old entire female Springer Spaniel diagnosed with Minimal Change Disease. The glomeruli did not reveal abnormalities histologically

**FIGURE 2:**
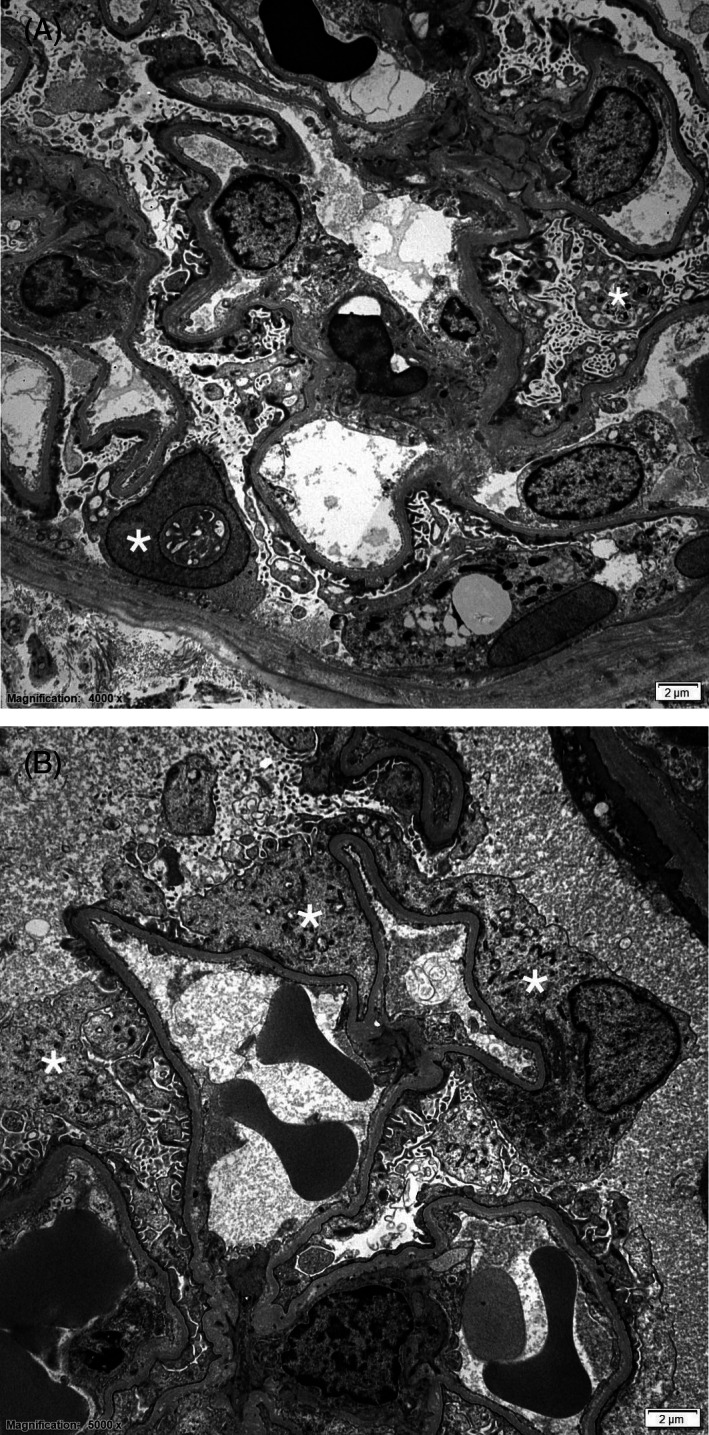
(A, B) Electron Microscopy showing marked effacement of the podocyte foot processes with microvillus transformation of podocyte cytoplasm. Podocytes are demarcated by asterisks (*) in a 3‐year‐old entire female Springer Spaniel diagnosed with minimal change disease. The capillary walls are of normal contour and thickness

Ultrasound‐guided renal biopsies were performed. The samples were considered good quality, containing 19 glomeruli of which none showed any abnormalities on light microscopy (Figure 1). The tubulointerstitial compartment was also normal. Immunofluorescence staining was performed on fresh frozen tissue as described^2^, ^10^, ^13^, ^14^, ^15^ and was negative for immunoglobulin heavy chains (gamma, mu, and alpha), lambda light chain, and the complement protein C3. Transmission electron microscopy of 3 glomeruli revealed moderate podocyte injury consisting of foot process effacement and microvillus transformation of the cytoplasm. There were no electron dense deposits observed in the glomerular capillaries or mesangial cells (Figure 2). Based on these findings, the dog was diagnosed with MCD of the podocytes. Treatment with telmisartan 1.25 mg/kg per os once daily (Semintra 10 mg/mL; Semintra 10 mg/mL; Boehringer Ingelheim Vetmedica GmbH, Rhein, Germany), mycophenolate mofetil 8 mg/kg per os every 12 hours (mycophenolate mofetil; Bova, London, UK) were initiated, and administration of prednisolone (0.3 mg/kg per os once daily [Prednidale; Dechra]) was continued.

The dog was reexamined every 3 to 4 weeks after starting treatment, and on each occasion was reported to be clinically well with no abnormalities detected on physical examination. Three weeks after starting treatment hypoalbuminemia and hypocholesterolemia had resolved. Urinalysis revealed a USG of 1.036, sediment analysis did not reveal abnormalities and the UPC was within normal limits (0.2 [ref <0.5]). The complete resolution of the proteinuria suggested an immune‐mediated etiology of MCD as described in human medicine^1^, ^2^; however, as the dog was also treated with angiotensin receptor blocker, we cannot exclude its effects on the resolution of the proteinuria. The dog was initially maintained on the same medication regimen for several weeks. At the subsequent reexaminations, serum albumin, cholesterol, and the UPC remained within reference intervals. The cholesterol was increased at 11 recheck, but no obvious reason was found. This was suspected to be secondary to the dog not being fasted at the time of the blood sampling, and the cholesterol was within normal ranges at subsequent rechecks. The dose of mycophenolate was gradually decreased over a period of 22 weeks before it was withdrawn. Prednisolone (Prednidale; Dechra) was continued for the treatment of the MUO; however, the dose was gradually decreased. Telmisartan (Semintra 10 mg/mL; Boehringer Ingelheim Vetmedica GmbH) was continued at the same dose. At the time of writing the dog continues to receive prednisolone 0.15 mg/kg per os every other day (Prednidale; Dechra) and telmisartan 1.25 mg/kg per os once daily (Semintra 10 mg/mL; Semintra 10 mg/mL; Boehringer Ingelheim Vetmedica GmbH). It remains clinically well and physical examination does not reveal abnormalities.

## 3 DISCUSSION

Minimal change disease has been rarely reported in veterinary literature. This could be because of the low prevalence, estimated around 0.6% in dogs,^10^ or the difficulty in detecting the disease if the tissue is analyzed only by light microscopy. The diagnosis requires electron microscopy to detect podocyte foot process abnormalities altering the integrity of the glomerular basement membrane.^10^, ^13^, ^14^, ^15^

In this case, the dog was suspected to be affected by idiopathic protein losing nephropathy. As proteinuria could indicate the presence of a systemic disease, a panel of diagnostic investigations was performed but no underlying cause was detected other than the MUO; however, the patient was treated for MUO for years without signs of proteinuria despite frequent monitoring and was believed in remission. Glomerulonephritis, glomerulosclerosis, and amyloidosis^10^, ^16^, ^17^ are the most common glomerulopathies in dogs; however, outcome and treatment are potentially different, and renal biopsies are necessary to make a definitive diagnosis.^17^, ^18^

The dog described in this case report was treated with long term prednisolone for meningoencephalitis of unknown origin. Glucocorticoids can cause glomerulopathies in dogs but typically the UPC is less severe, and hypoalbuminemia is typically not present.^19^ In this case, the proteinuria and hypoalbuminemia appeared to be a recent development as it was not previously observed despite blood tests and urinalysis being performed during the course of her MUO treatment. Examination of renal biopsy confirmed a diagnosis of MCD, and the glucocorticoids as the sole cause of the proteinuria was believed unlikely as histopathology of dogs on prednisolone therapy have been described in the majority of cases, with presence of mesangial hypercellularity, fusion of the foot process, occasional glomerular adhesion, and thickening of the Bowman's capsule.^19^, ^20^ As no trigger was found to induce the minimal change disease and with the history of MUO, MCD was believed to be immune‐mediated as in human medicine.^1^, ^2^, ^3^ In the majority of cases MCD in humans is considered to be idiopathic. However, a secondary MCD can be seen in adults and therefore potential underlying causes must be investigated and addressed.^15^, ^21^ The diagnosis of MCD in children is based on clinical suspicion and a response to treatment with corticosteroids. Renal biopsy is performed if no response is observed 4 weeks after initiation of treatment.^2^ In adults, the diagnosis is based on the histopathologic results, and corticosteroids are used as first line treatment if no underlying cause is identified. A complete remission of the proteinuria is expected within 16 weeks; however, in 10% to 20% of adults no response is observed,^2^ and repeat renal biopsy could be necessary to assess progression of the disease and evaluate for focal glomerular sclerosis.^2^, ^22^ In children and young adults, if there is no response to treatment with corticosteroids then a genetic form of MCD must be excluded.^2^ Other immune‐modulatory medications can be used as a second line treatment in patients with steroid‐resistant, steroid‐dependent, or relapsing disease; however, there is no strong evidence favoring the use of 1 agent compared to another.^1^, ^2^, ^23^, ^24^, ^25^ Minimal change disease occurs in dogs experimentally infected by *E. canis*, which resolves several weeks after inoculation without medical intervention.^5^ A Giant Schnauzer was diagnosed with MCD induced by masitinib, and the signs resolved after discontinuation of the drug.^7^ A 4‐year‐old, female Collie was reported idiopathic minimal change nephropathy with NS treated with antibiotics and diuretics; however, the dog was euthanized shortly after presentation and diagnosis of MCD was obtained only after necropsy.^6^ Mycophenolate mofetil was used in this case based upon recommendations from the ACVIM consensus statement for immune‐mediated glomerular disease,^26^ as MCD is believed to be an immune mediated disorders in humans.^1^, ^2^, ^3^ The dog showed complete resolution of the hypoalbuminemia, hypercholesterolemia, and proteinuria after initiation of mycophenolate mofetil and telmisartan, and was considered in complete remission of the podocytopathy. Because of the previous history of steroid‐responsive MUO, it was believed that an immune‐mediated etiology was more likely as a cause of the proteinuria, and that mycophenolate was mainly responsible of its resolution. However, as telmisartan was used concurrently it is also possible that this, or a combination of the 2 medications, was responsible for resolution of the proteinuria. In conclusion, this is the first case report of primary MCD podocytopathy successfully treated with mycophenolate mofetil and telmisartan in a dog. The remission of the hypoalbuminemia, hypercholesterolemia, and proteinuria was observed within 3 weeks after initiation of treatment and was maintained over the following 6 months.

## CONFLICT OF INTEREST DECLARATION

Authors declare no conflict of interest.

## OFF‐LABEL ANTIMICROBIAL DECLARATION

Authors declare no off‐label use of antimicrobials.

## INSTITUTIONAL ANIMAL CARE AND USE COMMITTEE (IACUC) OR OTHER APPROVAL DECLARATION

Authors declare no IACUC or other approval was needed.

## HUMAN ETHICS APPROVAL DECLARATION

Authors declare human ethics approval was not needed for this study.
